# Computed-Tomography-Guided Lung Biopsy: A Practice-Oriented Document on Techniques and Principles and a Review of the Literature

**DOI:** 10.3390/diagnostics14111089

**Published:** 2024-05-24

**Authors:** Lorenzo Saggiante, Pierpaolo Biondetti, Carolina Lanza, Serena Carriero, Velio Ascenti, Filippo Piacentino, Anas Shehab, Anna Maria Ierardi, Massimo Venturini, Gianpaolo Carrafiello

**Affiliations:** 1 Postgraduate School in Radiodiagnostics, Università degli Studi di Milano, 20122 Milan, Italy; lorenzo.saggiante@istitutotumori.mi.it (L.S.); carolina.lanza@policlinico.mi.it (C.L.); serena.carriero@policlinico.mi.it (S.C.); 2 Department of Diagnostic and Interventional Radiology, Foundation IRCCS Cà Granda–Ospedale Maggiore Policlinico, Via Francesco Sforza, 35, 20122 Milan, Italy; pierpaolo.biondetti@policlinico.mi.it (P.B.); annamaria.ierardi@policlinico.mi.it (A.M.I.); gianpaolo.carrafiello@unimi.it (G.C.); 3 Department of Diagnostic and Interventional Radiology, Circolo Hospital and Macchi Foundation, Insubria University, 21100 Varese, Italy; filippo.piacentino@asst-settelaghi.it (F.P.); massimo.venturini@uninsubria.it (M.V.); 4 Interventional Radiology Fellowship, Fondazione IRCCS Cà Granda, Ospedale Maggiore Policlinico, 20122 Milan, Italy; anas_shehab@hotmail.com; 5 School of Radiology, Università Degli Studi di Milano, Via Festa del Perdono, 7, 20122 Milan, Italy

**Keywords:** computed-tomography-guided lung biopsy, image-guided biopsy, percutaneous, lung cancer, pneumothorax, interventional radiology

## Abstract

Computed tomography (CT)-guided lung biopsy is one of the oldest and most widely known minimally invasive percutaneous procedures. Despite being conceptually simple, this procedure needs to be performed rapidly and can be subject to meaningful complications that need to be managed properly. Therefore, knowledge of principles and techniques is required by every general or interventional radiologist who performs the procedure. This review aims to contain all the information that the operator needs to know before performing the procedure. The paper starts with the description of indications, devices, and types of percutaneous CT-guided lung biopsies, along with their reported results in the literature. Then, pre-procedural evaluation and the practical aspects to be considered during procedure (i.e., patient positioning and breathing) are discussed. The subsequent section is dedicated to complications, with their incidence, risk factors, and the evidence-based measures necessary to both prevent or manage them; special attention is given to pneumothorax and hemorrhage. After conventional CT, this review describes other available CT modalities, including CT fluoroscopy and cone-beam CT. At the end, more advanced techniques, which are already used in clinical practice, like fusion imaging, are included.

## 1. Introduction

The technique of obtaining a sample of tissue from peripheral pulmonary lesions using a needle passing through an intercostal space, called percutaneous transthoracic lung biopsy (PTLB), was first described by Craver and Binkley (1939) [1]; the first lung core-needle biopsies (CNB) were described by Dutra and Geraci (1954) [2] and seemed promising for the diagnosis of focal and diffuse pulmonary disease [3,4].

In 1976, Haaga and Alfidi described the first cases of computed tomography (CT)-guided PTLB [5], after which, thanks to significant improvements in technique and equipment, this procedure reached a high degree of accuracy in diagnosing malignancy, both primary and metastatic.

## 2. Indications and Contraindications

Indications for CT-guided PTLB have changed over time, following changes in needle technology, immunohistochemistry, and imaging techniques.

Indications for PTLB, according to the Cardiovascular and Interventional Radiological Society of Europe (CIRSE) and the British Thoracic Society (BTS) guidelines [6,7], include the following:

- To establish the nature of diffuse parenchymal disease or persistent focal infiltrates without a definite diagnosis via sputum, blood cultures, serology, and bronchoalveolar lavage.
- To obtain material for microbiological analysis, particularly for infections refractory to standard treatments.
- To establish the nature, benign or malignant, of a suspected tumor, presenting as a new or enlarging mass or nodule or multiple nodules in a patient with no previous history of malignancy or who has had a prolonged remission.
- To classify a malignancy, including immunohistochemistry evaluation.
- To obtain material for molecular analysis.
- To stage a patient with known or suspected malignant tumor elsewhere.

In the management of lung nodules, current guidelines indicate the dimension of the lesion as the main criterion for biopsy, as it has been shown that the probability of malignancy increases proportionally with lesion’s size [8].

In particular, as reported by Gould et al., for nodules smaller than 8 mm, due to the low probability of malignancy and the difficulty in performing CNB, imaging surveillance with CT and/or PET with 2-deoxy-2-[fluorine-18]fluoro-D-glucose (^18^F-FDG PET) is recommended, with follow-up intervals evaluated based on additional criteria such as stability over time, clinical risk factors, and clinical judgment [9].

Nevertheless, when small nodules are found in oncological patients or in non-oncological patients with suspicious radiological features, with new or enlarging nodules, or with a high clinical risk, it may not be advisable to wait 3 or 6 months for the follow-up. The diagnostic accuracy of ^18^F-FDG PET-CT for nodules smaller than 10 mm is low, with a 19% false-negative rate in oncological patients [10]. Therefore, PTLB can also be considered for small nodules, being aware that in these circumstances, the procedure may be more challenging, resulting in a lower accuracy and a higher complication rate, with the possibility to obviate these problems thanks to technological advances, such as respiratory gating, C-arm cone-beam CT (CBCT), and CT fluoroscopy (CTF).

The absolute contraindications for PTLB, according to the CIRSE and BTS guidelines, are as follows:

- Failure to obtain consent.
- Lack of safe access.
- Non-correctable coagulopathy.

The relative contraindications, defined as conditions that increase the risk of complications and that, when possible, should be promptly corrected, are as follows:

- Coagulopathies.
- Uncooperative patients (general anaesthesia may be required).
- Significant comorbidities (hemodynamic or respiratory instability).
- Pregnancy (particularly if CT is required).
- Previous history of pneumonectomy (except for pleural lesions that can be approached without traversing the lung), vascular lesions suspected on CT, severe emphysematous disease, patients with intractable cough, and patients with pulmonary hypertension.

Other methods of obtaining lung tissue specimens include transbronchial lung biopsy (TBB), endobronchial ultrasound-guided transbronchial needle aspiration (EBUS-TBNA), and video-assisted thoracoscopic surgery (VATS). TBB uses flexible forceps positioned distally to obtain a piece of lung parenchyma, either blindly or with guidance from fluoroscopy, CT, or radial-probe endobronchial ultrasonography. In many cases, TBB can be a valid substitute to an open-lung biopsy, although for certain diagnoses, such as idiopathic pulmonary fibrosis, larger tissue samples are generally required.

TBB has proven useful in the diagnosis of sarcoidosis, Langerhans cell histiocytosis, Pneumocystis jirovecii pneumonia, and infections caused by Mycobacterium tuberculosis and pulmonary alveolar proteinosis. TBB also demonstrated a 70% yield for malignant peripheral lesions larger than 2 cm in diameter. An important role of TBB has also been demonstrated in lung transplant patients, helping in the diagnosis of rejection [11].

Because the pleura is not crossed, it is associated with a lower rate of pneumothorax (PNX) than PTLB, but the small size samples obtained are prone to crush artifact [12].

EBUS-TBNA is the procedure of choice to sample hilar and mediastinal adenopathies and is characterized by a minimally invasive nature and high sensitivity. In patients with non-small-cell lung cancer, EBUS-TBNA not only provides nodal staging but also allows for molecular testing, which is crucial for guiding the choice of therapy. It also shows a high diagnostic accuracy in patients with stages I and II sarcoidosis. Thanks to newer tools such as core needles and mini-forceps, larger specimens can be collected, increasing the diagnostic yield in patients with lymphoma and benign diseases and therefore reducing the need for more invasive procedures such as mediastinoscopy [13].

Finally, VATS is indicated for pleural biopsy, mediastinal lymph node biopsy, and biopsy of lung tissue in interstitial lung disease [14,15], being able to obtain larger samples of tissue but at a cost of an increased morbidity.

## 3. Main Types of Lung Percutaneous Image-Guided Needle Biopsy

**Figure 1:**
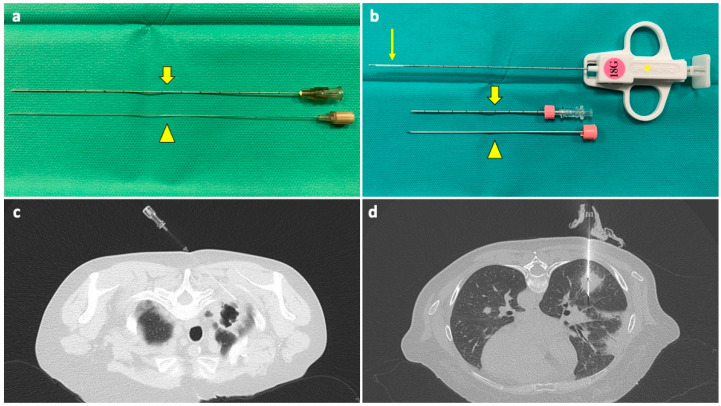
(**a**) A 22G FNA composed of an outer cannula (thick arrow) and an inner stylet (arrowhead); when in place inside the target lesion, the stylet is removed, and the cannula is connected to a syringe for aspiration. (**b**) A 18G CNB system composed of a coaxial needle, comprising an outer cannula (thick arrow), an inner stylet (arrowhead), and a biopsy needle (asterisk) with a sample notch at its tip (thin arrow); when the coaxial needle is in place, the stylet is removed, and the biopsy needle is inserted through the cannula. A biopsy gun activates a cutting cannula that moves over the sample notch, cutting the tissue inside it and allowing for collection. (**c**) The tip of a FNA needle has been positioned under CT guidance into the solid peripheral portion of an excavated lesion in the right upper lobe of a neutropenic 60-year-old woman with a history of breast cancer and a previous negative BAL. (**d**) The sample notch of a CNB needle is positioned under CT guidance into a solid mass in the lower right lobe of a 53-year-old neutropenic man with multifocal pneumonia, not responding to medical therapy, and with previous negative BAL. Abbreviations—BAL: bronchoalveolar lavage; CNB: core needle biopsy; CT: computed tomography; G: gauge.

Percutaneous needle biopsies of the lung can be represented by fine needle aspiration (FNA) or by CNB, depending on the needle type (Figure 1).

Whereas previously, the goal of the biopsy was to differentiate a malignant from a benign lesion, with general tumor subtyping if malignant (small-cell vs. non-small cell carcinoma), recently, this has evolved to include immunohistochemical analysis to diagnose adenocarcinoma, squamous cell carcinoma, or neuroendocrine carcinoma, as well as molecular profiling to identify gene mutations or immunotherapeutic biomarkers that may allow for target therapy. Therefore, today, the tissue obtained by the biopsy should be adequate not only for a diagnosis but also for the growing list of ancillary tests [16,17].

### 3.1. FNA

Aspiration needles are thin and flexible and are able to obtain specimens for cytologic or microbiologic evaluation. By convention, only needles ≥ 22 G can be defined as fine needles; they rely on the forward motion and the intrinsic capillary action of the needle.

The most commonly used is the Chiba (Cook, Inc. Bloomington, IN, USA), which has a 30-degree bevel and is available up to 25 G.

After reaching the target lesion, the central stylet is removed, a 10 mL syringe is attached, and suction is applied while rotating and slightly advancing and retracting the needle during suspended respiration. When material appears on the hub of the needle, suction is released. The presence on site of a cytotechnologist or cytopathologist is advised in order to extemporaneously evaluate the adequacy of the sample; if the sample is inadequate, FNA could be repeated or CNB could be performed.

### 3.2. CNB

Core needle biopsy is generally performed with a larger-gauge needle, ranging from 14 G to 20 G, which obtains a tissue core through a spring-loaded cutting action. A coaxial technique is usually adopted to allow for multiple sampling without increasing the number of pleural punctures.

The outer cannula should be advanced into the peripheral aspect of the target lesion.

When CNB is performed, it is important to confirm that the tip on the needle, once fired, remains located within the lesion or in a safe place.

CNB samples are placed directly in formalin for fixation, unless a lymphoproliferative disorder is suspected, in which case, a sample should be placed in normal saline or Roswell Park Memorial Institute (RPMI) solution for flow cytometry.

### 3.3. CNB vs. FNA

#### 3.3.1. Diagnostic Performance and Ancillary Testing

According to a metanalysis of approximately 20 studies, in the diagnosis of lung lesions, FNA and CNB had similar sensitivities (0.90 vs. 0.95) and specificities (0.99 vs. 0.99), respectively [18]. False-negative diagnoses are rare for both FNA and CNB [19]; however it is important to note that without a cytopathologist on site, the false-negative rate for malignancy has been demonstrated to be significantly higher with FNA [20].

FNA has been reported to have a false-positive rate of 0.8%, while no false-positive cases have been reported with CNB [21].

While the diagnostic accuracy of FNA for malignancy is similar to CNB [7,22], reaching up to 95% [23], its diagnostic accuracy for benign disease has been found to be lower [7,22,24,25,26,27]. It is important to note that the diagnostic accuracies of FNA vary widely, ranging from 64% to 97% [28,29], with high values achieved with large nodules [28,30,31] and an on-site cytopathologist [7,32,33]. The unavailability of a cytopathologist in many centers may lead the operator to choose CNB, particularly for small nodules.

Moreover, recent studies have also demonstrated that both methods provide specimens with comparably high adequacy rates for molecular testing [34,35]. However, a different study found that CNB provided a significantly higher percentage of samples adequate for molecular testing compared with FNA (67% vs. 46%, respectively) [36].

In general, for the diagnosis and ancillary testing of lung cancer, FNA and CNB appear to perform similarly, with some studies suggesting better molecular testing success with CNB.

#### 3.3.2. Complications

As for the reported complication rates, there is wide heterogeneity in the literature. Some studies report a higher complication rate for CNB compared to FNA [37,38], while others do not [20,39]. In a systematic review, Yao et al. concluded that no significant difference exist in terms of complication rate between the two techniques [40]. However, a more-recent metanalysis by Heerink et al. [41] found that CNB had a higher complication rate than FNA (38.8% versus 24.0%; *p* < 0.001), particularly for PNX, pulmonary hemorrhage (PH), and hemoptysis. For major complications, this difference was not significant, although a similar trend was noticeable.

For FNA procedures, larger needle diameter was a risk factor for overall complications, with the lower complication rate observed for an FNA needle gauge of 22 or higher [41]. Therefore, a 22 G needle is the commonly used needle for FNA.

Larger needles, potentially leading to more significant bleeding, may also dilute the extracted specimen, reducing its quality and adversely impacting the probability of making a diagnosis.

## 4. How Many Samples?

As for the number of passes, most operators usually perform at least two. Increasing the number of passes, the diagnostic accuracy of PTNB increases cumulatively, but the incremental gain reduces as the number of samples’ increases up to the third sample; between the third and fourth passes, no increase in sensitivity was found [42,43].

The number of passes performed is influenced by the difficulty of the procedure, the status of the lung parenchyma, the characteristics of the lesion, complications arising from each sampling, the quality of the specimens, and the need for cytological, histological, and microbiological analysis. The use of a coaxial needle allows for multiple samplings without repeating pleural and soft tissue puncture.

The presence of a cytopathologist during the procedure may reduce the number of passes [32,44,45,46].

The relationship between the number of passes and the complication rate has not been demonstrated, except for the finding that a higher number of pleural transgressions results in a higher rate of PNX [47].

## 5. Pre-Procedural Assessment

### 5.1. Coagulations Parameters

Prior to the procedure, the platelet count, international normalized ratio (INR), prothrombin time and activated partial thromboplastin time should be evaluated. According to guidelines, the procedure belongs to the “moderate/high risk of bleeding” group, for which a platelet count of >50,000/µL and an INR value of >1.5 are required. Major society guidelines contain indications on the need to suspend any anticoagulant or antiplatelet medication, as well as the exact timing to discontinue and restart the drug; for example, clopidogrel should be withheld for 3–5 days before procedure and restarted the day after, while prophylactic low-molecular-weight heparin should be withheld for 6–12 h before procedure and restarted 6–12 h after [48,49].

### 5.2. Pre-Procedural Imaging

Patients’ chest radiographs and CT scans should always be reviewed, if possible, at a multidisciplinary meeting. Imaging should be repeated if there is no recent imaging available, if the patient’s clinical condition has significantly changed, or if a significant change is revealed by the localizing CT scan at the time of the procedure. In particular, a chest CT is essential for pre-procedural planning because it can identify extrapulmonary lesions that may offer an alternative (and often preferable) site for biopsy, and it can help in selecting the more appropriate method of obtaining tissue specimens (PTLB, TBB, or VATS) [7,50].

In the case of multiple pulmonary lesions, chest CT guides the choice of the target lesion, which is based on size and location, with larger and more superficial lesions being generally easier; an exception are juxtapleural lesions which, despite being the most superficial, may be challenging approach percutaneously because of respiratory motion, particularly in the lower lobes [51].

CT is also useful in selecting the appropriate imaging guidance modality, in selecting the patient’s position and needle type, and in planning the best needle trajectory based on the lesion location and the surrounding bronchovascular structures, fissures, and bullae, keeping in mind that the length of the parenchyma crossed should be minimized [52]. As for contrast administration, the advantages of pre-procedural contrast-enhanced CT images include the demonstration of the nature of the target lesion, the definition of any cystic or necrotic components, and the detection of significant vessels within the lesion. ^18^F-FDG PET is useful in identifying the metabolically active portion of a pulmonary lesion, which needs to be targeted after visual or digital images coregistration to improve accuracy [53,54].

### 5.3. Immediate Pre-Procedural Checklist

A 4–6 h fasting prior to the procedure is recommended, particularly when sedation is planned. Peripheral venous access should be obtained, and vitals should be monitored during the procedure. Antibiotic prophylaxis is not routinely recommended.

It is crucial that the patient cooperates during the procedure; therefore, biopsies should be performed without sedation whenever possible [7], which makes a precise explanation before the procedure (particularly focusing on the potential stinging sensation during the pleural puncture and on how to suspend respiration) and an adequate local anesthesia even more important. Occasionally, an oral anxiolytic drug can be helpful; moderate sedation can be useful for elderly patients with difficulties lying still due to pain, for children, and for non-cooperative patients. It is important to avoid over sedation because it can result in irregular respiration.

## 6. Imaging Guidance

The imaging modality for biopsy guidance is selected based on the preprocedural CT scan, and the choice depends on lesion size and location and preference of the operator. In the great majority of cases, CT, in the form of conventional CT, CTF, or CBCT, is the method of choice. Although it is not the focus of this review, ultrasound guidance is also possible but limited to subpleural lesions abutting the chest wall; ultrasound has been described as safe and fast and has the advantages of being less expensive and of avoiding exposure to ionizing radiation [55].

Radiation exposure should be monitored and minimized as much as possible. As stated before, intravenous contrast medium, though not routinely administered, can be helpful in differentiating the lesion from atelectasis, viable from necrotic tissue, and in better delineating vascular structures. 

## 7. Conventional CT

### 7.1. Patient Positioning

Patient positioning (prone, supine, or lying on the side) is chosen based on the planned access site, which depends on lesion location and is also influenced by the patient’s tolerance of the position. When possible, the prone position is preferred because the intercostal spaces are wider posteriorly than anteriorly, the posterior portion of the ribs moves less, and because the needle is not seen by the patient, with possible benefits in terms of anxiety; however, this position may not be tolerated by older patients and caution must be used in cases of low respiratory function. When using the supine position, arms may be kept alongside the body to increase patient stability and comfort. The side and oblique positions are less stable, but they can be useful for approaching lateral subpleural lesions. When needed, arms can be raised above the head to widen the intercostal spaces or, in prone position, to move the scapulae laterally, possibly facilitating needle trajectory.

### 7.2. Breathing

Respiratory motions must be taken into consideration when planning the access route, and patients should be instructed according to the operator’s needs. In general, respiratory excursion is greater in the lower lobes. Some operators prefer to perform the biopsy procedure with the patient breathing freely as this implies some degree of movement of the target lesion but avoids the variability of deep breath-holds, which can be a source of even wider movements in poorly cooperative patients. On the other hand, breath-holding is preferred by other operators during image acquisition and needle movements, with the goal of obtaining the same degree of lung volume and thus a stable anatomy throughout the procedure. An option is to ask the patient to hold their breath after deep expirations or inspirations, though it can be difficult in cases of concomitant diseases, stress, or fatigue; another option is to ask the patient to breath normally, avoiding wide respiratory excursions, and to try to hold their breath more or less at the same point of the respiratory cycle. Respiratory gating techniques can also be used, and their use has been demonstrated to improve procedural accuracy for small nodules [56].

### 7.3. Procedural Steps

Once the patient is positioned on the table, the first CT scan is performed. While limiting the scan in craniocaudal extent has a substantial impact on the total procedural radiation dose [57], obtaining a first CT scan which includes the entire lung volume may be important for choosing the most appropriate target. On the first scan, the axial slice containing the desired entry site is chosen and displayed on the patient’s skin through the gantry axial laser light. Radiopaque markers, usually in the form of grids, are placed across the laser light perpendicularly. After performing a second CT scan with a smaller acquisition volume centered on the chosen axial slice, the entry site is defined as the intersection between the gantry light and one of the radiopaque markers; the point is then marked with a dermographic pen on the patient skin. After sterilization of the skin entry site, local anesthesia is administered, paying attention not to reach the pleura with the needle to avoid PNX. The biopsy needle is inserted directly through the skin or after a small skin incision. The procedure consists of progressive advancements and adjustments of the needle from the entry point to the target lesion, first in subcutaneous tissues and then in lung parenchyma; each modification in the needle position is controlled with one CT acquisition, always of a small volume centered on the procedural site (the so-called “biopsy mode”). It may be useful to use a specific protocol with automatic reconstruction of images in the sagittal plane when an “off axis” route is used. The operator usually walks out of the CT suite during acquisition, with radiation release controlled at the CT console; after reviewing the acquired images, the operator advances or adjusts the needle and then repeats these steps until the target has been reached. It is preferrable to spend more time achieving a good angle of the needle into the subcutaneous tissues before pleural puncture, rather than performing a higher number of needle adjustments when the needle is already in the lung parenchyma.

Pleural puncture should be performed in a single sharp movement, with the needle oriented perpendicularly to the pleural surface; repeated pleural punctures should be avoided. The needle should pass through the shortest possible tract of normal lung parenchyma, avoiding risky structures such as fissures and bullae. Attention must also be paid to avoid major vessels both in the chest wall and in the lung; entrance in the lower part of the intercostal space is preferred to avoid intercostal nerves and vessels.

Once the intervention phase of the procedure has been completed, a final CT scan is performed to exclude early adverse events such as hemorrhage or PNX.

Following the biopsy, the patient should be monitored in a recovery area for a minimum of 1–2 h; then a upright chest radiograph should be performed to rule out PNX [7,52]. The majority of PNX are detected 1 h after the procedure [58,59].

## 8. Complications

Risks factors for complications of PTNB are summarized in Table 1.

### 8.1. Pneumothorax

Pneumothorax can develop from two mechanisms: either by communication through the chest wall, or from the lung by rupture of the visceral pleura [66].

It is the most common complication that occurs during or immediately after a PTLB, with an estimated rate of 12–45%, and a consequent chest tube placement rate of 2–15% [67]. PNX that develops 3 or 4 h after needle lung biopsy has been defined as “delayed” [68].

A tension PNX occurs when the pressure in the pleural space is positive throughout the breathing cycle, resulting in decreased venous return, hypotension, and hypoxia [66].

#### 8.1.1. Risk Factors

One of the factors that more strongly influences PNX rate is target tumor depth, with different results in the literature. From Yeow et al.’s [69] series of 660 PTLB, lesion depth was the single more significant predictor of PNX, with the lower risk occurring in lesions abutting the pleural surface and the highest risk in subpleural lesions between 1 mm and 20 mm in depth, with a decrease in PNX rates with a further increase in depth; this finding leads to the conclusion that the risk of PNX is strongly correlated only with the violation of the aerated lung. The authors suggested that in order to prevent inadvertent dislodgement of the guiding needle, a longer, oblique needle path might be advisable, as also advised by others [70,71,72]. Khan et al. [62] reported that the PNX rate was significantly higher when the lesions were located in the lung parenchyma compared with pleural lesions (*p* < 0.05), but all PNX cases requiring chest tube placement occurred in lesions located less than 2 cm from the pleura.

Different results were found by Moulton et al. [73], who, using the coaxial technique, reported an increased risk of PNX in subpleural lesions, especially those abutting the pleura surface, with a possible explanation being that a short needle path does not provide sufficient anchoring during respiratory movements, with consequent possible dislodgement of the coaxial cannula into the pleural cavity, causing air ingress and pleural tears.

As for FNA, the increased traversed lung parenchyma was a risk factor for complications, including PNX [41].

Regarding the size of the lesion, smaller lesions are technically challenging, requiring longer procedure times and potentially more needle adjustments, which can result in higher complication rates [52]. Cox et al. demonstrated a PNX rate of 58.5% for lesions of 2 cm or smaller, compared to 30.9% for lesions larger than 2 cm [61]. However, other investigators who used the coaxial technique demonstrated no increase in PNX rate for smaller lesions [20,74]. Also, in the case of FNA, smaller lesion size was found to be a risk factor for complications including PNX [41].

Chronic obstructive pulmonary disease (COPD) raises the PNX rate from 7% to 47% [52,75], and emphysema is a strong predictor of chest tube placement (OR 4.01) [76].

Regarding needle size, as stated before, there is no concordance in the literature [38,39], but Heerink et al. reported a lower PNX rate for FNA compared to CNB and, among FNA, a lower PNX rate for smaller needles [41].

Some studies demonstrated that a small angle between the needle trajectory and the pleural surface is an independent risk factor of PNX [77,78,79]; the pleural hole produced by a needle inserted at a shallow angle may be larger than that produced by a needle inserted perpendicularly to the pleura.

According to some studies, a lower lobe location of the target lesion is a risk factor for PNX [65,78], whereas lesions in the upper and middle lobes increase the risk of chest tube placement if PNX occurs. This is mainly due to the more pronounced respiratory motion of the lower lobe, which makes the procedure more challenging, demanding more needle redirections and increasing procedural time.

As for the timing of PNX onset, delayed PNX has been found to be more frequent in upper lobes [80,81], and the explanation could be that the less aerated upper lobes result in later onset of PNX.

Radiologist experience represents another major factor impacting PNX rate, with a 17% PNX rate for biopsies performed by experienced radiologists compared to 30% for less-experienced radiologists [52].

#### 8.1.2. Prevention

Several techniques that could potentially reduce the risk of PNX have been investigated. During the procedure, positioning the patient in the lateral decubitus with the biopsy-side-down has been reported to reduce the PNX rate [82,83].

If the procedure has been performed in a supine or prone position, rapidly positioning the patient with the puncture side-down (rapid roll-over) has been shown to reduce the rate of PNX and of chest tube placement [84]. Biopsy-side-down posture may improve visceral and parietal pleura adhesion and cause alveolar size reduction, creating a physical barrier to additional air leakage [85].

Deep expiration and breath-holding during needle extraction has proven capable of reducing PNX and the chest tube insertion rate by 50% [86], probably because of positive intrapleural pressure.

After removing the biopsy needle, creating a blood patch with the infiltration of autologous venous blood along the needle track has been shown to reduce the risk of PNX requiring drainage [87,88]; the blood can be withdrawn beforehand from the intravenous line.

The effectiveness of the instillation of other materials, such as compressed collagen foam plugs, fibrin glue, normal saline, and hydrogel plugs, as a sealant has been investigated [76,78,79,80]. These substances can be injected into the coaxial needle while withdrawing it to fill the biopsy tract, creating a barrier that may prevent air from being sucked from the alveoli into the pleural space [88].

After the procedure, patients should refrain from talking, moving, and coughing as much as possible in order to avoid increases in intrathoracic pressure.

If a PNX occurs during or following a lung biopsy, the air should be immediately aspirated from the pleural space prior to removal of the needle, obtaining reapposition of visceral and parietal pleura (pleural patching), which may prevent progression of PNX, reducing the need for chest tube placement [89].

A protocol combining biopsy-side-down patient positioning, needle removal during expiration, autologous blood patch sealing, rapid rollover, and pleural patching (PEARL) has been proved to reduce the rates of PNX and chest tube insertion, with a similar complication rate to TBB [90].

#### 8.1.3. Management

The management of PNX depends on the severity of clinical symptoms and on the size of the PNX [85]. In general, observation and oxygen supplementation are recommended for patients with limited stable PNX without symptoms [91,92]. Guidelines also recommend that patients with a small PNX without breathlessness should be considered for an early discharge, with written advice to return to the hospital if breathlessness occurs [91].

**Figure 2:**
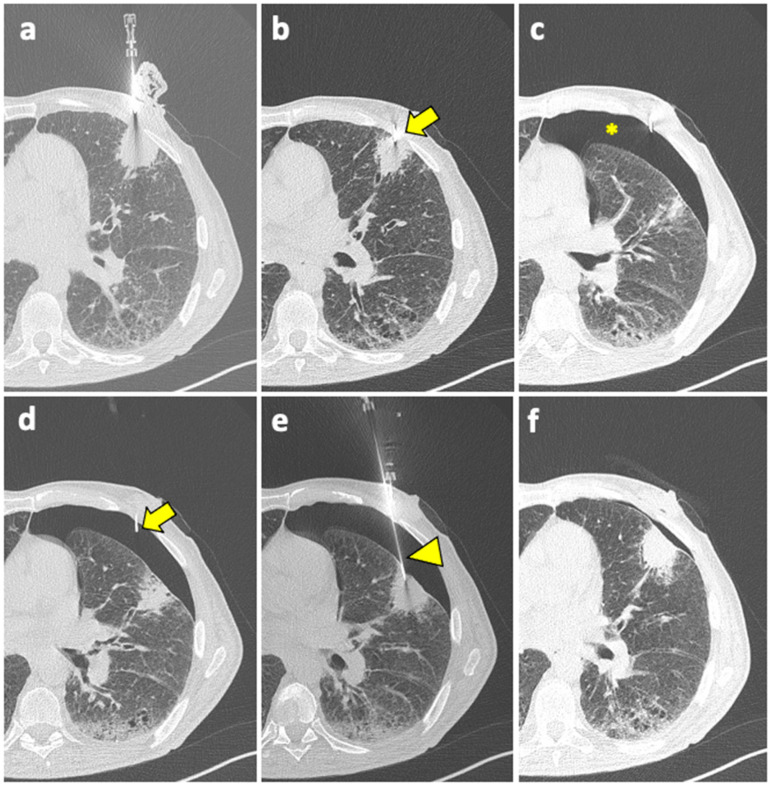
(**a**) A coaxial needle is pointed under CT guidance towards a solid mass in the left upper lobe of a 61-year-old man with history of COPD, smoking, and follicular lymphoma. (**b**) The tip of the needle (arrow) is pushed forward into a juxta-plural position. (**c**) At the following CT scan, pneumothorax is detected (asterisk). (**d**) The coaxial needle is pushed slightly forward into the air space (arrow), the stylet is removed, and the cannula is connected to a syringe for manual aspiration. (**e**) At the same time, a new, longer coaxial needle (arrowhead) is positioned into the target lesion across the pneumothorax, and a core needle biopsy is performed (histology: diffuse large-B-cell lymphoma). (**f**) Final CT scan after manual aspiration shows significant reduction of the pneumothorax; patient had stable vitals during follow-up and did not require a chest tube insertion. Abbreviations—COPD: chronic obstructive pulmonary disease; CT: computed tomography.

Patient with moderate–severe PNX and/or breathlessness require active intervention, with simple aspiration being suggested as initial treatment [91]. If still in place, the coaxial cannula used for the biopsy can be retracted back into the pleural space and used for aspiration; otherwise, it can be reinserted near the biopsy site. An example of manual aspiration is shown in Figure 2.

Manual aspiration is efficient at preventing PNX advancement because it creates an apposition between the visceral and the parietal pleura. When PNX develops despite manual aspiration, a chest tube is needed [85]; a small-bore catheter (10–14 F) should be placed for chest drainage [84,91].

The coaxial biopsy needle can be used to access the pleural space and to place a chest tube via the Seldinger technique. Otherwise, the tube can be inserted by the interventional radiologist in the anterior second or third intercostal space under CT guidance.

In cases of large, recurrent, or tension PNX, or in patients with clinical instability characterized by severe dyspnea and hypoxemia [85,92], a chest tube is required immediately. A 16-F-to-22-F chest tube should be inserted in patients who are not mechanically ventilated and not at high risk for a significant air leak, while for patients receiving mechanical ventilation, a larger-bore chest tube (>24 F) should be taken into consideration to handle possibly substantial air leakage [93].

### 8.2. Pulmonary Hemorrhage

**Figure 3:**
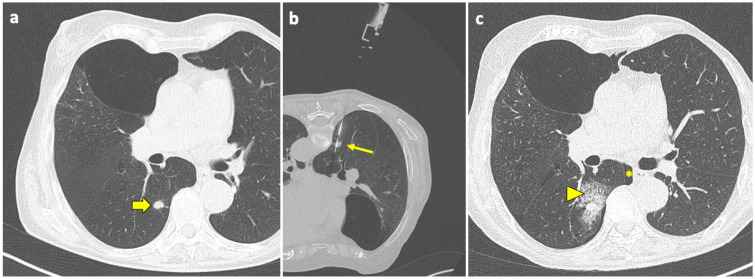
Post-procedural perilesional hemorrhage. (**a**) New solid pulmonary nodules are discovered during follow-up of a 73-year-old woman with a history of breast and rectal cancer, including one in the right lower lobe (thick arrow). (**b**) With the patient in a prone position, the sample notch of a CNB system (arrow) is positioned under CT guidance at the level of the nodule, and biopsy is performed. (**c**) At the end of the procedure, the patient is turned into the supine position and a final CT scan is performed, which shows a minor-degree pneumothorax (asterisk), along with a ground-glass opacity and septal thickening in the tissue around the nodule, consistent with localized hemorrhage (arrowhead). Both complications had no evolution or sequelae. Abbreviations—CNB: core needle biopsy; CT: computed tomography.

Pulmonary hemorrhage is the second most common complication after PTLB, with a prevalence ranging from 4 to 27% [94]. In the majority of cases, this complication is mild and only detected on the post-biopsy CT scans as ground-glass opacity (GGO) around the lesion or along the needle tract, representing an alveolar hemorrhage in clinically asymptomatic patients, as shown in Figure 3.

Typically, no treatment is required, and the only measure that should be adopted is to position the patient with biopsy-side-down to avoid the aspiration of blood into the unaffected lung. When PH becomes symptomatic or a higher-grade PH arises, oxygen and pro-coagulative therapy may be required.

The risk factors for PH include lesion size, lesion depth, pulmonary hypertension [94,95], lesion size < 2 cm and lesion depth > 2.1 cm in particular [69], and CNB [41].

On the other hand, patients with PH along the needle track were two times less likely to develop PNX, suggesting that the presence of blood may prevent air passage from the alveolar space and/or promote adhesion between visceral and parietal pleura. The same authors also reported a protective role of emphysema against bleeding, likely due to the paucity of vessels [96].

Chest wall hemorrhage, mediastinal hemorrhage, and hemothorax are extremely uncommon and severe complications resulting from the inadvertent passage of the needle through a variety of arteries, including internal thoracic, intercostal, or mammary arteries [97]. Current guidelines [98] are in favor of draining hemothoraxes of any size, although there is substantial evidence to suggest that minor hemothoraxes can be managed conservatively with favorable outcomes [99,100,101].

### 8.3. Air Embolism

Air embolism is a rare but life-threatening complication of PTLB, consisting of the passage of air within the systemic circulation through a pulmonary vein. Even a small quantity of air in the coronary or cerebral arteries can have a devastating effect, causing serious morbidities and death [102], with a reported sequelae rate of 11.2% and a death rate of 21.5% in patients with symptomatic air embolism [103], with the most common symptoms being a comatose/unresponsive state, hemiplegia, hypotension, and cardiovascular arrest [104].

The incidence of air embolism after PTLB is reported to range between 0.06% and 4.80% [104], and this high variability may be representative of the number of asymptomatic undiagnosed air embolisms [105,106].

As to the genesis of this phenomenon during PTLB, one possible mechanism plays out when the tip of the coaxial needle is in a pulmonary vein and the inner stylet is removed, allowing for communication between the atmosphere and the vessel, while the other mechanism involves the post-puncture formation of a bronchovenous or alveolus-venous fistula [107,108].

The operator must instruct the patient to suspend respiration when the coaxial system is open and to avoid coughing and deep inspiration as much as possible throughout the procedure.

The methods often employed to prevent the passage of atmospheric air, therefore reducing the risk of gas embolism, are the use of sterile saline during and after the removal of the internal stylet [109], the use of the operator’s own finger, or the reinsertion of the stylet after removal of the biopsy needle from the cannula.

When air embolism is suspected, the patient should immediately start receiving 100% high-flow oxygen and should be positioned in the right lateral decubitus and in the Trendelenburg position to avoid passage of air to the left ventricular outflow tract, which has a trend toward more favorable outcomes [103].

### 8.4. Tumor Seeding

The retrograde spread of tumor cells along the needle’s path is known as “tumor seeding”. For lung biopsies, the incidence of seeding is smaller in comparison to other districts, such as the liver [110], and the use of the coaxial technique further reduces the risk [111,112]. For instance, Kim et al. reported 8 cases out of 4365 procedures. Additionally, following a successful resection, the patient’s prognosis appears to be solely based on the underlying malignancy, regardless of the chest wall implantation [113].

The only exception is for lesions adjacent to the pleura, where the risk of pleural recurrence was higher in patients who had undergone biopsy [114].

## 9. CT Fluoroscopy

CTF guidance combines the high anatomical definition of CT with the convenience and safety of real-time fluoroscopic imaging. The operator works in the CT suite for the entire duration of the procedure, using a control panel covered with a sterile transparent casing to manage the CT scan. It can be performed either with continuous CTF or intermittent (also known as sequential, quick check, or step-and-shoot) CTF. While in continuous CTF, images are acquired at a frequency of 7-to-12 frames/second as long as the foot pedal is pressed; in intermittent CTF, the operator acquires one or more contiguous slices, limiting real-time imaging to short glimpses to visualize the position of the needle tip. The acquisition of several (usually three, up to as many as six) contiguous slices is the most commonly used method, enabling us to visualize the tip of the needle and at least one image above and one image below. The operator can then adjust the needle and re-acquire images for sequential adjustments, quickly alternating between live imaging and needle movement [115,116].

Another useful feature is the gantry tilt, which allows the procedure to be carried out at a slight angle (maximum 30°) in the craniocaudal plane.

When compared to conventional CT, CTF has shown significantly higher diagnostic accuracy in a recent meta-analysis [117]. CTF, allowing for real-time monitoring of lesions’ movement, also results in fewer needle passes and adjustments, shorter procedure times, and lower complication rates [118,119].

However, this comes at the cost of a significantly higher radiation exposure to both patient and operator, especially for continuous CTF [120,121,122,123]: Kim et al. reported a mean surface dose to patients of 34.9 mSv for the CTF group and of 18.7 mSv for conventional CT, as well as a mean DLP of 384.0 mGy × cm and 160.1 mGy × cm, respectively [27]. Therefore, continuous CTF is rarely used today, and should be reserved for complicated lesions, including small lesions, lesions near critical structures, or lesions that move widely with respiration.

As for intermittent CTF, its CT dose index (49.3 mGy) was more than 10-fold lower than the continuous CTF group (561.6 mGy) [120].

## 10. Cone-Beam CT

CBCT-virtual-navigation-guided lung biopsy has been developed in the last decade.

**Figure 4:**
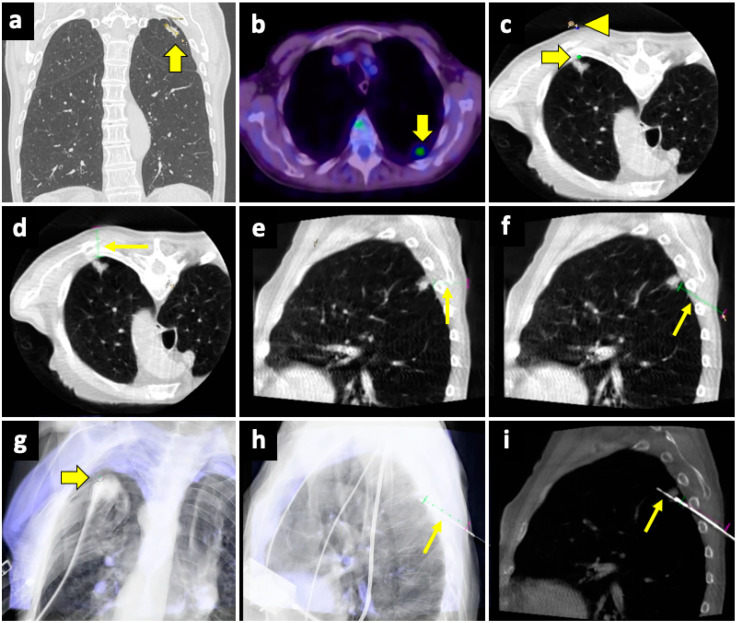
Biopsy, pneumothorax, and manual aspiration. (**a**) A left upper lobe solid lesion (thick arrow) is detected in a 77-year-old man with COPD. (**b**) The lesion demonstrates FDG avidity at the PET-CT exam (thick arrow), and the tumor board gave instruction to simultaneously conduct biopsy and ablation, which were performed under CBCT guidance. (**c**) After acquiring the first CBCT scan, the target point (thick arrow) and the entry point (arrowhead) are selected at the workstation using the dedicated software (Philips XperGuide). (**d**) A virtual trajectory (thin arrow) is created between the target and entry points; note that the axial trajectory passes through the scapula and a rib in the axial view. (**e**) The impossibility of using an axial trajectory (thin arrow) is also confirmed in the sagittal view. (**f**) The entry point is changed, and the new oblique virtual trajectory (thin arrow) now passes through intercostal space. (**g**) The established virtual trajectory is displayed over the real-time fluoroscopic images in the “entry point” view, in which the entry and target points correspond (thick arrow); this view is used in this case to introduce the devices (microwave antenna and coaxial needle system) from the skin towards the target. (**h**) Visualization can occur also in a plane perpendicular to the prior one, the “progression” view, in which the virtual trajectory (thin arrow) can be seen at the maximum of its length; this view is used to check the progression of the devices along the established trajectory. (**i**) The Sagittal CBCT image shows the sample notch of a CNB system positioned at the level of the target lesion (thin arrow). The lesion was then biopsied (histology: lung adenocarcinoma) and ablated. Abbreviations—CBCT: cone-beam-computed tomography; FDG: fluorodeoxyglucose; PET-CT: positron emission tomography-computed tomography.

The CBCT system consists of flat-panel fluoroscopy and a CBCT scanner. The execution of CBCT-virtual-navigation-guided lung biopsy is performed in steps. The first step is to acquire a CBCT scan for adequate planning of the biopsy. Briefly, using the software (various software exist, by different vendors), the operator establishes the target lesion and the needle entry point at the skin surface, after which a virtual segment connecting the entry and target points is shown, representing the needle path. On the basis of this planning, the software automatically calculates the correct C-arm position to display the needle entry point on the patient’s skin. This phase of the procedure is called “entry point positioning”. After skin disinfection of the access area, under fluoroscopic guidance, the operator positions the needle tip at the cutaneous entry point, based on the CBCT image appearing on the monitor as a fusion of the 3D volume previously acquired using CBCT and the real-time fluoroscopy bidimensional plane. At this time, the C-arm rotates in the “progression view” position, perpendicular to the previous one. Then, the needle is advanced into the chest to reach the target point, following the virtual path previously determined and displayed on the monitor in real-time fluoroscopy. Then, a second CBCT scan is performed to verify the needle’s correct placement, and the biopsy is taken. A final scan is acquired to assess any complications [124]. An example of CBCT-guided biopsy is illustrated in Figure 4.

This technique overcomes the limits of conventional CT, the lack of real-time monitoring during the puncture, and the lack of flexibility due to the forced choice of the axial plane [65,125,126], allowing for a real-time fluoroscopy visualization of the needle puncture and of the solid lesion movement due to respiration, as well as a free 3D rotation of the C-arm around the patient, leading to the best puncture path [127,128].

CBCT-virtual-navigation-guided lung biopsies are safe and accurate, with a high technical success rate (>99%) and high accuracy (>95%) [127,129]. Moreover, compared to conventional CT guidance, CBCT virtual navigation facilitates needle positioning and reduces the procedural time, the effective X-ray dose [125,129], and the incidence of complications [125]. Moreover, in solid lung lesions smaller than 15–20 mm, its accuracy was higher compared to conventional CT guidance (98.2% vs. 83.7%) [128]. Good results using CBCT guidance in small pulmonary nodules (<1 cm) were also reported by Choo et al., with sensitivity, specificity, and diagnostic accuracy of 96.7%, 100%, and 98%, respectively, with only a 6.5% rate of PNX and 5.6% of hemoptysis [130]. Using CBCT, no significant differences in sensitivity, specificity, and accuracy between the PTLB of nodules <1 cm and larger nodules (from 1 to 2 cm) were found [131].

## 11. Advances and New Techniques: PET/CT-CBCT Fusion Imaging

The possibility that the biopsy may not be diagnostically conclusive is a concern not only for smaller nodules but also for large masses.

In fact, within such lesions, as their size increases, this also increases the possibility that not all the tissue is vital and that necrotic areas may be present, which, if sampled, would yield non-diagnostic or false-negative results; it is reported that lesions between 5 cm and 10 cm often need a rebiopsy, which would likely lead to an increased complication rate [132].

**Figure 5:**
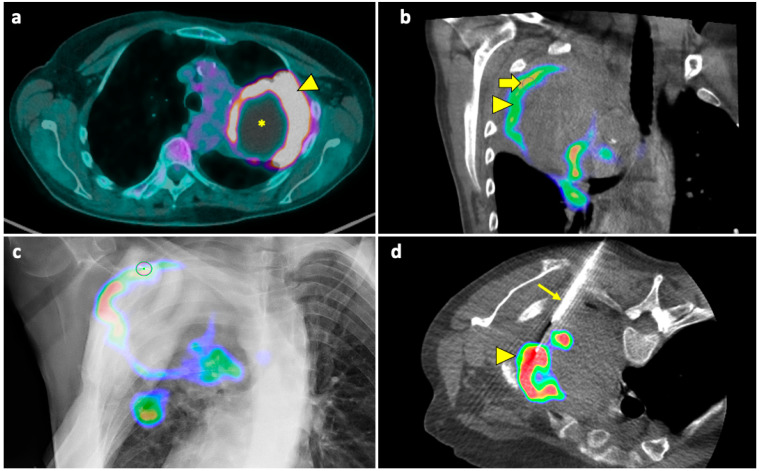
Biopsy guided by fusion imaging between CBCT and ^18^F-FDG PET-CT. (**a**) Preprocedural PET-CT showing a large mass in the upper lobe of the left lung, with a metabolically active peripheral area (arrowhead) and a central photopenic area (asterisk). (**b**) Coronal fused image obtained by overlapping the intraprocedural unenhanced CBCT volume to the preprocedural PET-CT volume; note that the mass has no distinguishable components on the CBCT images. Fusion imaging allows us to identify the metabolically active portions (arrowhead) and to place the target point (thick arrow) within this area. (**c**) The procedural PET-CT volumetric data and the virtual trajectory are displayed over the real-time fluoroscopic images as guidance during the biopsy. (**d**) Axial fused image obtained by overlapping the intraprocedural CBCT to the preprocedural PET-CT images shows that the biopsy needle (thin arrow) is in the right direction, i.e., towards the most metabolically active part of the mass (arrowhead). Histology revealed pulmonary adenocarcinoma.

A useful technique in this setting is fusion imaging, which is defined as the process of overlapping imaging datasets from different modalities into a single composite imaging dataset. By using dedicated software, it is possible to overlap pre-procedural ^18^F-FDG PET/CT images to intra-procedural CBCT images, thus integrating functional and anatomical data, and to guide the needle towards the viable and metabolically active portion of the target lesion. An example of a biopsy guided by fusion imaging between CBCT and PET-CT is illustrated in Figure 5.

It has been demonstrated that the technique is effective [133], increasing the chances of obtaining diagnostic samples while performing fewer biopsies, improving the quality of histological analysis [134].

## 12. Advances and New Techniques: Spectral CT-CBCT Fusion Imaging

Another option is to register intraprocedural CBCT images to dual-layer spectral CT images. Spectral CT is a novel technology that enables better tissue characterization, specifically using *Z*-effective images based on the effective atomic number of tissue components, potentially us allowing to detect more-viable neoplastic tissue areas to be biopsied [135]. Information on the effective atomic number of the tissue is obtained from the spectral decomposition in the form of photoelectric and Compton scattering data [136]. The atomic number of a tissue is also correlated with the presence of iodine [137].

Areas with higher *Z* values are highly vascularized, and Curti et al. hypothesize that, by using *Z*-effective images to guide biopsy, it could be possible to avoid poorly vascularized areas, which are potentially less diagnostic due to necrosis and fibrosis.

Spectral CT, with its ability to provide enhanced tissue characterization and differentiation, holds significant promise as a guiding tool for biopsies in the future, potentially improving accuracy, diagnostic yield, and patient outcomes. In the near future, spectral CT could improve interventional procedures and may emerge as an optimal tool for planning and guiding biopsies, aiming to minimize the risk of obtaining false-negative or non-diagnostic samples and to reduce the number of rebiopsies [138].

**Figure 6:**
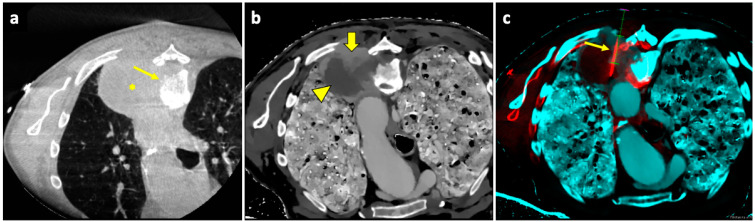
Biopsy guided by fusion imaging between CBCT and ^18^F-FDG PET-CT. (**a**) Axial intraprocedural CBCT image with the patient in prone position reveals a large lung lesion (asterisk) in the apical segment of the left lower lobe, causing erosion of the adjacent vertebral body (thin arrow); no significant difference in cellularity within the lesion can be appreciated in the unenhanced CBCT. (**b**) Axial CT image reconstructed with spectral Z-effective grayscale map demonstrates significant tissue differentiation within the lesion, allowing for identification of a portion with high cellularity (thick arrow) and another component with low cellularity and high fibrous tissue content (arrowhead). (**c**) Intraprocedural fusion image of CBCT and Z-effective images with the needle inserted in the cellular portion (thin arrow). Abbreviations—CBCT: cone-beam-computed tomography; CT: computed tomography.

An example of a biopsy guided by fusion imaging between pre-procedural spectral CT and intra-procedural CBCT is illustrated in Figure 6.

## 13. Lung Biopsy and Thermal Ablation

Lung biopsy is usually required along with thermal ablation of lung lesions in inoperable patients. Asynchronous biopsies are performed as separate procedures which occur before the ablation treatment session. The other option is to perform synchronous biopsy and ablation during the same session.

In asynchronous biopsies, the confirmation of pathological diagnosis avoids unnecessary treatments. At the same time, asynchronous procedures expose the patient to two high-risk procedures and increase the duration of hospital stay and costs.

The advantage of performing biopsy and ablation at the same time is to expose the patient to the risk of complications only once, decreasing hospital stay and costs [138].

Nevertheless, in simultaneous procedures, PNX and PH induced by biopsy may affect the feasibility and efficacy of subsequent ablation, particularly in GGO from PH, which may reduce the visibility of the target lesion [139].

## 14. Conclusions

Percutaneous CT-guided lung biopsy is a safe and highly accurate method for the diagnosis of indeterminate focal pulmonary lesions. Careful preprocedural planning, the choice of the most appropriate guidance modality, and adherence to strict procedural steps, with knowledge of the pitfalls and the risk-lowering techniques, can result in low complication rates and successful outcomes. This review aims to offer a comprehensive view of all the information that may be needed when approaching percutaneous lung biopsy, from pre-procedural assessment and choice of imaging guidance, including recent advances (i.e., fusion imaging and spectral CT), to the technical aspects of the procedure and its possible complications, discussing their management and methods that may reduce them.

## Figures and Tables

**Table 1:** Risk factors for complications.

	OVERALL COMPLICATION	PNX	PNX Int	PH
Needle type (CNB vs. FNA) [41]	*p* < 0.001 (39% vs. 24%)	*p* = 0.027 (25% vs. 19%)	-	*p* = 0.005 (18% vs. 6%)
FNA caliber (<22 G) [41]	*p* < 0.001	-	-	-
Lesion size (<2 cm)	*p* < 0.001 [60]	*p* ≤ 0.001 (59% vs. 31%) [47,61]	*p* = 0.012 [47]	*p* = 0.006 [62], *p* = 0.015 [63]
Lesion depth	*p* < 0.001 [60]	*p* < 0.0001 [62], *p* = 0.0097 [63]	*p* < 0.001 [47]	*p* < 0.0001 [60,61]
Emphysema	*p* = 0.001 [60]	*p* < 0.001 (41% vs. 24%) [64]	*p* < 0.01 [41], *p* < 0.001 (27% vs. 9%) [47]	Protective (*p* = 0.024) [47]
Lower lobes	-	*p* = 0.002 [65]	-	-
Number of pleural punctures	0.008 [60]	*p* < 0.001 [47]	*p* = 0.002 [47]	*p* < 0.01 [62]

Abbreviations—CNB: core-needle biopsy; FNA: fine needle aspiration; PNX: pneumothorax; PNX int: pneumothorax requiring intervention; PH: pulmonary hemorrhage.
